# Dopamine D1-Like Receptor Stimulation Induces CREB, Arc, and BDNF Dynamic Changes in Differentiated SH-SY5Y Cells

**DOI:** 10.1007/s11064-024-04293-8

**Published:** 2024-11-27

**Authors:** Omar B. Rivera-Maya, Christian D. Ortiz-Robles, José R. Palacios-Valladares, Emma S. Calderón-Aranda

**Affiliations:** Department of Toxicology, Center for Research and Advanced Studies of the National Polytechnic Institute, Mexico City, Mexico

**Keywords:** Dopamine D1-like receptor, Arc, Brain-derived neurotrophic factor, cAMP response element-binding, Protein Kinase A

## Abstract

The dopamine D1-like receptor is a dopamine (DA) receptor regulating diverse brain functions. Once the dopamine D1-like receptor is activated, it induces activation of the Protein Kinase A (PKA) that phosphorylates the cAMP Response Element-Binding (CREB) transcription factor, which once active elicits the expression of the critical synaptic elements Activity-regulated cytoskeleton-associated (Arc) and the Brain-Derived Neurotrophic Factor (BDNF). The temporality and subcellular localization of proteins impact brain function. However, there is no information about the temporality of CREB activation and Arc and BDNF levels induced through dopamine D1-like receptor activation. In this study, we aimed to assess the specific effect of dopamine D1-like receptor activation on the temporality of CREB-phosphorylation (p-CREB^S133^) and the spatiotemporal induction of Arc and BDNF. Using SY-SY5Y cells differentiated with Retinoic Acid (RA), the dopamine D1-like receptor activation with a specific agonist transiently increased p-CREB^S133^ at 30 min of stimulation. It induced two spikes of Arc protein at 15 min and 6 h, forming clusters near the cell membrane. BDNF secretion temporarily increased, reaching a maximum at 6 h, while secretion was lower at 24 h compared to the unstimulated group. Our results provide new insight into the role of dopamine D1-like receptor activation on CREB activation, Arc, and BDNF increase, showing that these effects occur temporally and for Arc in subcellular specific sites. This study highlights the dopaminergic system as a critical regulator of subcellular events relevant to neuron plasticity. Future research should address the study of the implications for brain function and behavior.

**Supplementary Information:**

The online version contains supplementary material available at 10.1007/s11064-024-04293-8.

## Introduction

Distinct neurotransmitters involve brain functions such as cognition and emotional or behavioral responses. One of these is the dopaminergic system, which through dopamine (DA), operates through five receptors, subdivided into two families: the dopamine D1-like receptors, constituted by Dopamine Receptor D (DRD)1 and DRD5, and the D2-like receptors, constituted by DRD2, DRD3, and DRD4 [1]. It has been established, through deletions and pharmacological tools, that the dopamine D1-like receptor actively participates in the development of memory, learning, and attention [2, 3]. However, the underlying mechanisms still need to be fully established. The dopamine D1-like receptor activation induces a cascade reaction including the cyclic adenosine monophosphate (cAMP) production and activation of cAMP-dependent Protein Kinase A (PKA), triggering several responses encompassing transcription, translation, and post-translational modifications of different synaptic players [4]. PKA activates the cAMP Response Element-Binding (CREB) transcription factor by phosphorylation in Ser^133^ (p-CREB^S133^), which is necessary for CREB to bind at the gene promoter region to initiate gene transcription [5]. p-CREB^S133^ induces transcription of the Immediate Early Genes (IEGs), such as the *Arc* gene, coding for the Activity-regulated cytoskeleton-associated (Arc) protein, and the *BDNF* gene, coding for the Brain-Derived Neurotrophic Factor (BDNF) [6]. The Arc protein (Arg3.1) regulates the trafficking of glutamate receptors and participates in actin polymerization dynamics to modify synaptic structures [7, 8]. BDNF functions include the regulation of signaling pathways involved in survival and synaptic events [9]. It has been suggested that the function of these proteins, in addition to the expression and activation, requires a specific spatiotemporal location [10, 11]. Scarce evidence exists on the specific responses elicited by the dopamine D1-like receptor’s activity on temporal activation of CREB and expression of Arc and BDNF. Growing evidence suggests that different abnormalities in brain functions are related to alterations not only in protein levels but also in post-translational events like location, transport, recycling, secretion, endocytosis, or second messengers’ stability, contributing to the development of neurodegenerative conditions like Lafora’s, Alzheimer’s, and Parkinson’s disease [12, 13].

Regarding the above, we aimed to assess the specific effect of dopamine D1-like receptor activation on the temporality of p-CREB activation and the spatiotemporal induction of Arc and BDNF in SH-SY5Y cells previously differentiated with Retinoic Acid (RA).

## Materials and Methods

### Culture of SH-SY5Y Cells and Differentiation

The SH-SY5Y neuroblastoma cell line (CRL-2266 from ATCC, Manassas, VA, USA) was used in passages 1 to 15. Cells were cultured in DMEM/F12 medium (GIBCO-BRL, Grand Island, NY, USA) supplemented with 10% Fetal Bovine Serum (FBS) (PAN-Biotech, Aidenbach, GER), 1% sodium pyruvate (Sigma-Aldrich, St Louis, MO, USA), 1% L-glutamine (Sigma-Aldrich, St Louis MO, USA), 1% non-essential amino acids (Sigma-Aldrich, St Louis MO, USA), and 1% penicillin-streptomycin (Sigma-Aldrich, St Louis, MO, USA). Cultures were maintained at 37 ºC, a CO_2_ atmosphere (5%), and a humid environment. Cell differentiation was performed according to previous studies, with minor modifications [14, 15]. Briefly, the cells were seeded at 30,000 cells/cm^2^ density, stabilized for 24 h, and treated with RA 10 µM (Sigma Aldrich, St Louis, Missouri) dissolved in ethanol with 2% FBS for seven days. The supplemented media was renewed every other day. Cell differentiation was confirmed by the assessment of neurites emerging from the cells. Using a phase contrast microscope (Olympus FSX100), images of differentiated cells in culture were taken, and the neurite length was determined using Image J software (NIH, USA). Additionally, the expression of Synapsin (Syn), a specific neuronal marker, was evaluated by Western blot (see below).

### Cell Treatments

Once the cell differentiation was confirmed, the cells were stimulated with 10 µM of (±)-SKF-38393 (Sigma-Aldrich, St Louis, MO, USA) (only referred to as SKF-38393 in the text body), a specific agonist of dopamine D1-like receptor for 0.25 (15 min), 0.5 (30 min), 1, 6, and 12 h. Exceptionally, for BDNF determination, the time of SKF-38393 stimulation was extended to 24 h. To determine whether effects on proteins of interest depend specifically on dopamine D1-like receptor activation, 10µM of (R)-(+)-SCH-23390 (Sigma-Aldrich, St Louis, MO, USA) (only referred to as SCH-23390), a specific antagonist of the dopamine D1-like receptor, was added 30 min before starting the stimulation with SKF-38393.

### Cell Viability Assay

Cell viability was determined through mitochondrial activity by the 3-(4,5-dimethyl-2- thiazolyl)-2,5-diphenyl tetrazolium bromide (MTT) colorimetric assay (Sigma-Aldrich, St. Louis MO, USA), according to Mosmann (1983) [16]. The absorbance of MTT was evaluated at 590 nm, and the viability percentage was calculated and compared to controls.

### Western Blot Detection of Syn, DRD1, Arc, BDNF and p-CREB^S133^

The protein levels of DRD1, Syn, Arc, and BDNF were determined in total protein extracts. After treatment, cells were lysed and processed, as previously reported [17]. The protein level p-CREB^S133^ was assessed in the nuclear extract: cells were lysed with 0.1% Nonidet P40 in Buffer A and processed, as previously reported [18]. The Bradford method was used for protein quantitation. Homogeneous quantities (20–40 µg/well) of protein from each experimental condition were loaded and separated by sodium dodecyl sulfate (SDS)-polyacrylamide gel electrophoresis (PAGE). Proteins were electro-transferred to a polyvinylidene fluoride (PVDF) membrane (Millipore Corporation, Bedford, MA, USA) in a Hoefer semidry transfer unit (Hoefer Pharmacia Biotech Inc, San Francisco, CA, USA). PVDF was blocked with 5% non-fat dry milk in PBS containing 0.1% Tween-20 (PBS-Tween) for 1 h. Membranes were incubated overnight at 4 °C with antibodies against Syn (1:1000), DRD1 (1:1000), Arc (1:500), BDNF (1:1000), p-CREB^S133^ (1:500), β-actin (1:5000), and Lamin B1 (1:500) (Santa Cruz Biotech., Santa Cruz, CA, USA). β-actin and Lamin B1 antibodies were used as a total and nuclear loading control, respectively. Membranes were washed three times with PBS-Tween 1% and incubated with horseradish peroxidase (HRP)-conjugated to anti-goat Ab (1:3000) (Santa Cruz Biotech., Santa Cruz, CA, USA) or HRP-conjugated anti-mouse Ab (1:3000) (Bio-Rad, Hercules CA, USA) for 2 h at room temperature. After washing three times with PBS-Tween, bands were visualized using Western blotting luminol reagents (Bio-Rad, Hercules CA, USA) detecting chemiluminescence signal by the photo-documenter Fusion FX (Vilber, Marne-la-Vallée, FR). Densitometric analyses were performed using ImageJ software (NIH, USA).

### Immunofluorescence Assay for Arc and p-CREBS133 Proteins Cell Location

SH-SY5Y cells were seeded at 20,000 cells/cm^2^ density and differentiated onto Nunc™ Lab-tek™ II chamber slides (Thermo Scientific, Waltham, MA, USA). The staining protocol was according to what had already been reported [19]. Cells were stained with mouse anti-Arc or mouse anti-p-CREB^S133^ antibodies (Santa Cruz Biotech., Santa Cruz, CA, USA) (both at 1:30). The secondary antibody fluorescein isothiocyanate (FITC)-conjugated anti-mouse-IgG (Thermo Scientific, Waltham, MA, USA, 1:300). The cytoskeleton was stained with 1:250 tetramethyl rhodamine B isothiocyanate (TRITC)-phalloidin (Sigma-Aldrich, St. Louis, MO, USA). Nuclei were detected with 1:500 of 4′, 6-diamidino-2-phenylindole (DAPI) (Sigma-Aldrich, St. Louis, MO, USA). The cells were mounted in Vectashield mounting medium (Vector Laboratories, Burlingame, CA, USA), and fluorescent images were captured with a confocal laser microscope (TCS SP8 DM6000, Leica Microsystems).

### Detection of BDNF Secretion

The BDNF-secreted and intracellular protein levels were determined in cell culture supernatant media using a quantitative antigen capture Enzyme-linked immunosorbent assay (ELISA) against the mature form of BDNF. The ELISA assay was performed according to the manufacturer’s instructions for Duo Set Kits (R&D Systems, Minneapolis, MN, USA).

### PKA Activity Assay

The PKA activity was quantified in cell lysate using a PKA Kinase Activity Kit (Enzo Life Sci., Inc., Farmingdale, NY, USA) according to the manufacturer’s instructions. After differentiation and treatments, cells were lysed, and the Bradford method was used to quantify the protein concentration. Then, 0.1 µg of whole protein per well was loaded onto the PKA Substrate Microtiter Plate after following the manufacturer´s instructions. Briefly, the PKA in cell lysates was activated by adding ATP to phosphorylate to the specific peptide substrate previously adhered to the plate. A specific antibody was added to phospho-specific substrate detection, and subsequently, a peroxidase-conjugated antibody and the substrate tetramethylbenzidine were used. The reaction was stopped with an acid solution, and the absorbance was read in a spectrophotometer at 450 nm. The formula for calculating the PKA activity was:

$$\eqalign{& {\text{Relative Activity Kinase }} = \cr & {{{\text{Average absorbance of sample }} - {\text{ Average absorbance of blank}}} \over {{\text{Quantity of crude protein used per assay}}}} \cr} $$

### Statistical Analysis

After determining data normality using the Shapiro-Wilk test, the data were analyzed using the one-way or two-way ANOVA tests followed by the Bonferroni or Tukey *post hoc* test as established in figure legends. The student’s t-test was used to compare two groups (the control versus a specific treated condition), as indicated in figure legends. The data are expressed as the mean ± SEM of three independent experiments. The p-value < 0.05 was considered statistically significant. Statistical analysis was performed using Graph Pad Prism 5.0 software (Graph Pad Software, Inc., San Diego, CA, USA).

## Results

### Morphological and Molecular Changes in SH-SY5Y Cells Differentiated with RA

**Fig. 1:**
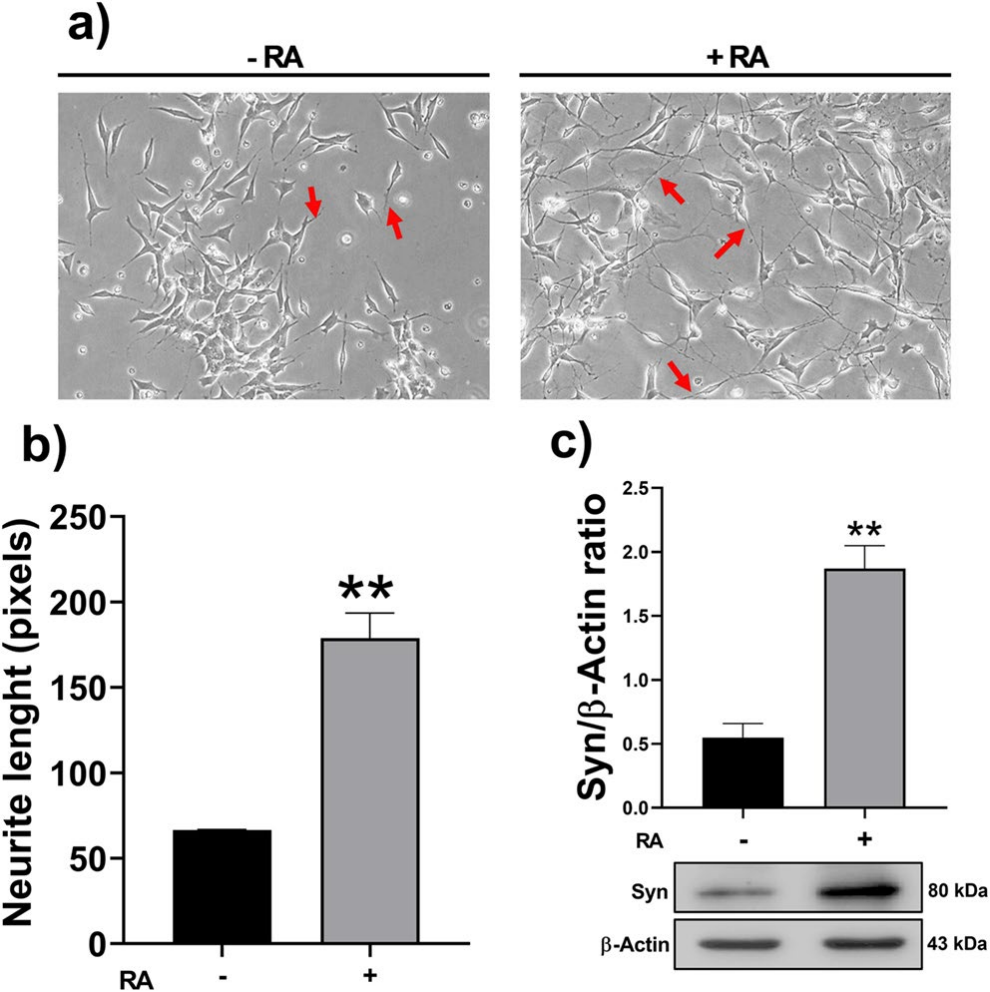
Morphologic and molecular changes after seven days of differentiation with RA in SH-SY5Y cells. **a**) representative micrographs showing the emergent neurites (red arrow) of the SH-SY5Y cells treated (right) and untreated (left) with RA; **b**) each bar represents the neurite length mean ± SEM (*n* = 4 cells/per micrograph by triplicate) determined in pixels from soma to the end of each process from three independent experiments. **c**) A representative immunoblot of the Syn and β-Actin expression is shown; each bar represents the mean ± SEM of the Syn/Actin ratio from three independent experiments. A Student´s *t*-test was used to determine differences induced by RA treatment; ** *p* < 0.01 indicated statistical difference compared RA treatment to w/o treatment

The SH-SY5Y human neuroblastoma cells (with a well-known ability to be differentiated to like-neuronal phenotype) were subjected to seven days of differentiation process treatment with RA 10 µM. After, morphological and molecular changes in RA-treated cells were compared with the untreated cells to confirm differentiation. The RA-treated cells show neurite formation (Fig. 1a), characterized by long processes (like-filopodia structure) arising from the cell soma, showing a significant augment of neurite length compared to the untreated cells (Fig. 1b). Additionally, the RA-treated cells have a higher expression of Syn, a molecular marker for mature neurons, compared to the untreated cells (Fig. 1c). These results confirm the SH-SY5Y cell differentiation to neuronal-like phenotype after treatment with RA.

### Effects of RA-induced SH-SY5Y Differentiation on Levels of DRD1, PKA, p-CREB^S133^, Arc, and BDNF

**Fig. 2:**
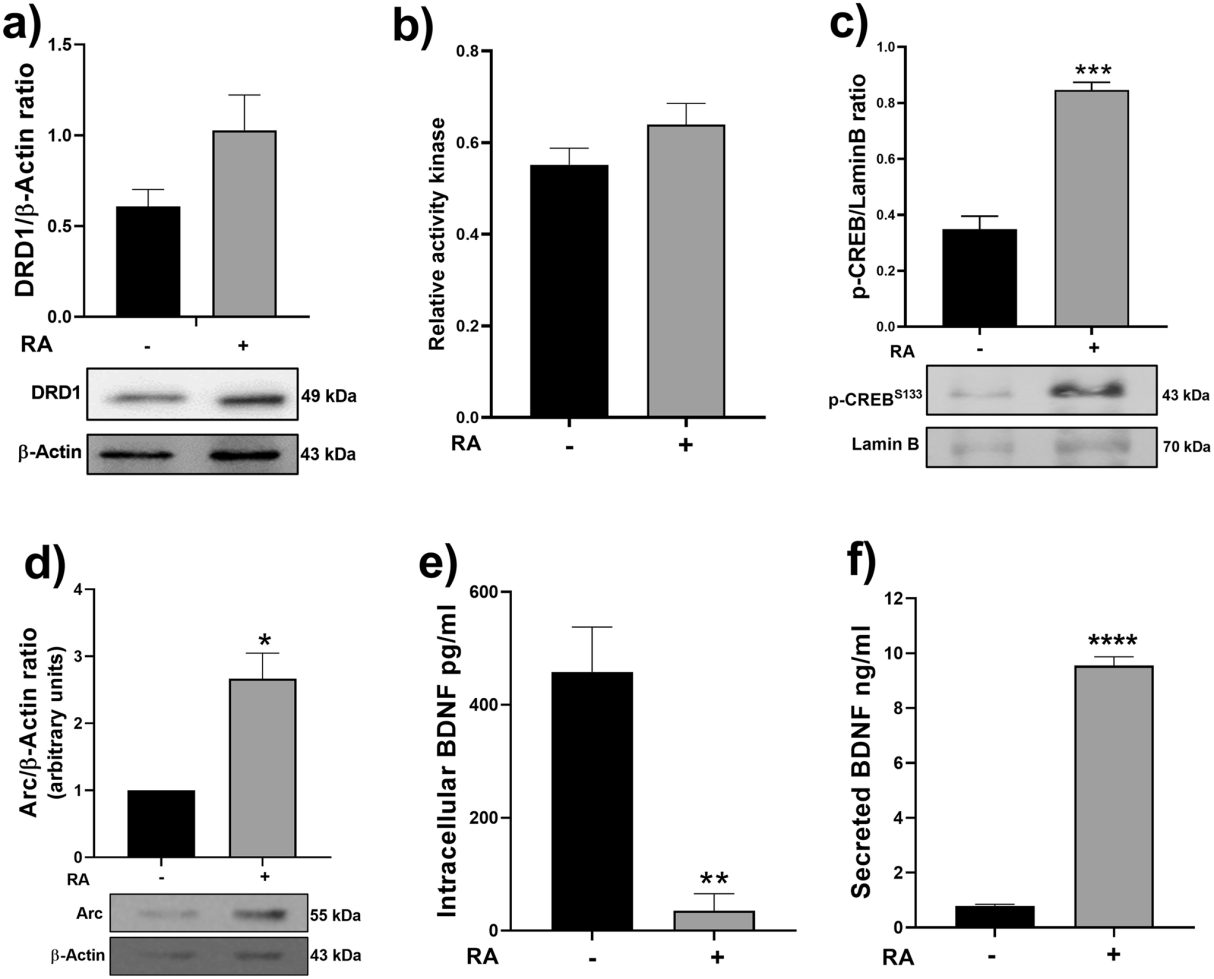
Effect of the RA-induced differentiation on DRD1 level, PKA activity, p-CREB^S133^, Arc, and BDNF intracellular level/secretion in SH-SY5Y cells. In cultures treated or not with RA (10µM) were determined: **a**) The DRD1 level (immunoblot at the bottom); each bar represents the mean ± SEM of DRD1/β-actin ratio from three independent experiments. **b**) The PKA activity level; each bar represents the mean ± SEM of Relative activity kinase from three independent experiments in duplicates. **c**) The nuclear expression level of p-CREB^S133^ (immunoblot at the bottom); each bar represents the mean ± SEM of p-CREB^S133^/Lamin B ratio from three independent experiments, ***p < 0.001 statistical difference compared RA treatment to w/o treatment. **d**) The expression level of Arc (immunoblot at the bottom); each bar represents the mean ± SEM of Arc/β-actin ratio from three independent experiments, *p < 0.05 statistical difference compared RA treatment to w/o treatment. The BDNF (**e**) intracellular and (**f**) secreted level, each bar represents the mean ± SEM of BDNF from three independent experiments by duplicate, ** *p* < 0.01 and **** *p* < 0.0001 statistical differences compared RA treatment to w/o treatment. Data were analyzed by t-student test

The levels of DRD1 (a member of the dopamine D1-like receptor), p-CREB^S133^, Arc, and BDNF proteins, and the PKA activity were evaluated in RA-treated and untreated cells. No difference in DRD1 protein levels (Fig. 2a) and PKA activity (Fig. 2b) was observed between both groups. The p-CREB^S133^ and Arc levels increased significantly in RA-treated compared to untreated cells (Fig. 2c, d). The intracellular BDNF levels in RA-treated cells were lower than in the untreated cells (Fig. 2e). However, in the culture’s supernatant, a significant increase of BDNF secreted by RA-treated cells compared to baseline levels in untreated cells was observed (Fig. 2f). These results confirm that RA-induced SH-SY5Y differentiation triggers the increase of p-CREB^S133^, Arc, and the BDNF secretion, without affecting the DRD1 level nor the PKA activity. Considering this evidence, the subsequent experiments were performed only in RA-treated cells.

### Effects of the Dopamine D1-like Receptor Activation in RA-treated SH-SY5Y Cells on DRD1 Protein Level and PKA Activity

**Fig. 3:**
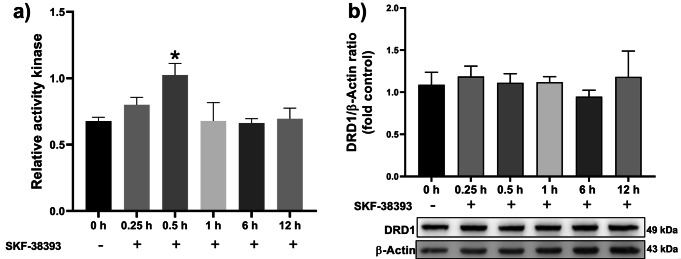
Effects of dopamine D1-like receptor activation on PKA activity and DRD1 level. **a**) the PKA activity was determined at indicated times of stimulation with SKF-38393 (10µM); each bar represents the mean ± SEM of Relative activity kinase from three independent experiments by duplicated, * *p* < 0.05 statistical difference compared cells stimulated with SKF-38393 (0.5 h) to cells w/o stimulation (F = 3.071). **b**) A representative immunoblot result of DRD1 and β-Actin is at the bottom; each bar represents the mean ± SEM of DRD1/β-Actin ratio from three independent experiments (F = 0.2998). Data were analyzed using a one-way ANOVA and Tukey´s *post hoc* tests

Since the dopamine D1-like receptor activation triggers the cAMP/PKA signaling [4], to determine whether the treatment with SKF-38393 activated this DA receptor, the PKA activity level was evaluated after 0.25, 0.5, 1, 6, and 12 h. Treatment with SKF-38393 transiently increased the PKA activity at 0.5 h compared to the non-activated control (Fig. 3a). With this result, it was hypothesized that the DRD1 level changes by its activation with SKF-38393. However, no modification occurred in DRD1 levels after stimulation at all times evaluated (Fig. 3b). The induction of the PKA activity by the agonist of the dopamine D1-like receptor suggests that these receptors are functional in RA-treated SH-SY5Y cells.

### The Dopamine D1-like Receptor Activation Transiently Induces CREB^S133^ Phosphorylation, Arc, and BDNF

**Fig. 4:**
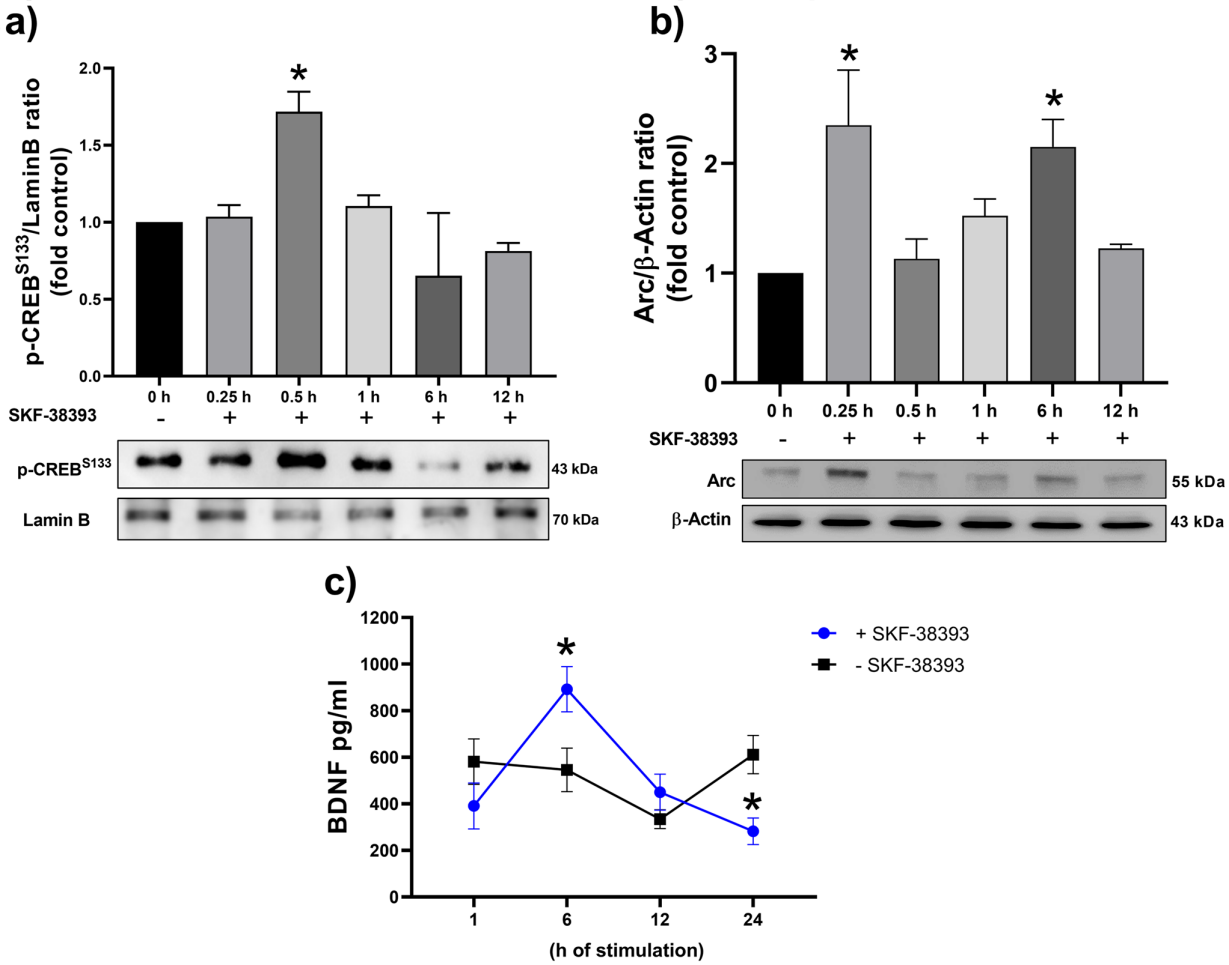
Effects of dopamine D1-like receptor activation on p-CREB ^S133^level,Arc level,and BDNF secretion. **a**) A representative immunoblot of p-CREB^S133^ and Lamin B is at the bottom of the graphic; each bar represents the mean ± SEM of the p-CREB^S133^/Lamin B ratio from three independent experiments, * *p* < 0.05 statistical difference compared cells stimulated with SKF-38393 (10 µM) (0.5 h) to cells w/o stimulation (F = 6.517). **b**) A representative immunoblot of the Arc and β-actin level is at the bottom of the graphic. Each bar represents the mean ± SEM of the Arc/Actin ratio from three independent experiments, * *p* < 0.05 statistical difference compared cells stimulated with SKF-38393 (0.25 and 6 h) to cells non-stimulated (F = 4.378). **c**) ELISA determined the BDNF secretion; each bar represents the mean ± SEM of three independent experiments by duplicated, * *p* < 0.05 statistical difference compared cells stimulated with SKF-38393 (6 and 24 h) to cells w/o stimulation. Results in **a**) and **b**) were analyzed using a one-way ANOVA test and Dunnet´s *post hoc* tests. Results of **c**) were analyzed using a two-way ANOVA test and Tukey´s *post hoc* test

The RA-treated cells were stimulated with SKF-38393 for 0.25, 0.5, 1, 6, and 12 h to evaluate the effects of activation of dopamine D1-like receptor on the level of p-CREB^S133^, the level and location of Arc, and secretion of BDNF. The BDNF secretion was also evaluated at 24 h. The nuclear p-CREB^S133^ level significantly increased at 0.5 h after the dopamine D1-like receptor activation compared to the non-activated control (Fig. 4a). The dopamine D1-like receptor activation induced two spikes of the Arc protein compared to the non-activated control; an early spike was observed at 0.25 h and a late one was observed at 6 h (Fig. 4b). The dopamine D1-like receptor stimulation induced a significant increase in BDNF secretion at 6 h compared to non-activated control (Fig. 4c). In contrast, after 24 h of dopamine D1-like receptor stimulation, the level of BDNF secretion was lower than in the non-activated control. As the BDNF secretion reached the maximum levels at 6 h of dopamine D1-like receptor activation, this time was chosen to be quantified in the subsequent experiments.

### A Specific Antagonist of Dopamine D1-like Receptors Prevented the Effect Induced by Dopamine D1-like Receptor Activation on CREB, Arc, and BDNF

**Fig. 5:**
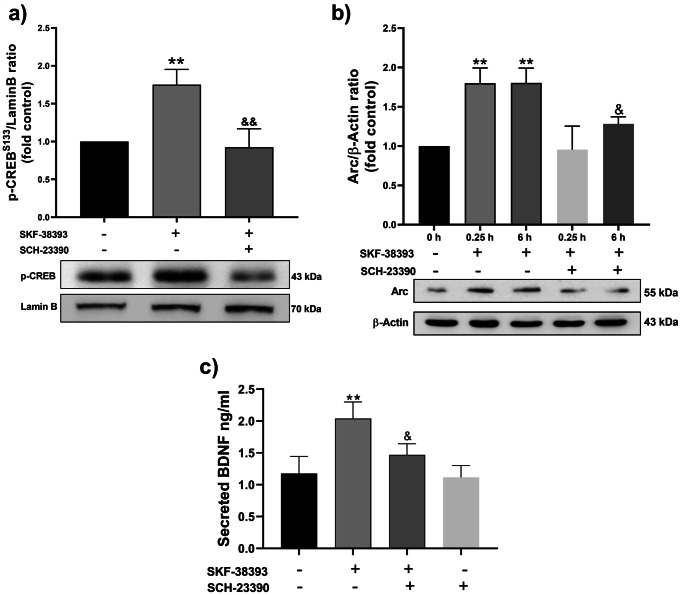
Blocking the dopamine D1-like receptor inhibits the Arc, BDNF, and p-CREB ^S133^ induction. After the pretreatment with antagonist SCH-23390 (10µM), the cells were stimulated with agonist SKF-38393 (10 µM) at maximum induction time for each parameter. **a**) A representative immunoblot of p-CREB^S133^ and Lamin B levels is at the bottom of the graphic; each bar represents the mean ± SEM of p-CREB^S133^/Lamin B ratio from three independent experiments, ** *p* < 0.01 statistical difference compared cells stimulated with SKF-38393 (0.5 h) to cells w/o stimulation, and ^&&^*p* < 0.01 cells treated with SCH-23390 + SKF-38393 to cells stimulated with SKF-38393. **b**) A representative immunoblot of Arc and β-Actin level is at the bottom of the graphic; each bar represents the mean ± SEM of the Arc/β-Actin ratio from three independent experiments, ** *p* < 0.01 statistical difference compared cells stimulated with SKF-38393 (0.25 and 6 h) to cells w/o stimulation, and ^&^*p* < 0.05 statistical difference compared cells treated with SCH-23390 + SKF-38393 (6 h) to cells stimulated with SKF-38393 (6 h) (F = v4.564 for 0.25 min and F = 14.98 for 6 h). **c**) The BDNF secretion level determined by ELISA, each bar represents the mean ± SEM from three independent experiments by duplicated, * *p* < 0.05 statistical difference compared cells stimulated with SKF-38393 to cells w/o stimulation (F = 11.52). Data were analyzed using a *t*-test or one-way ANOVA test followed by a Tukey *post hoc* test

A selective antagonist of the dopamine D1-like receptor (SCH-23390) was used before stimulating with SKF-38393 to evaluate whether the CREB, Arc, and BDNF inductions depended on dopamine D1-like receptor activation. In the presence of the antagonist SCH-23390, significant inhibition of the SKF-38393-induced nuclear level of p-CREB^S133^ (Fig. 5a), the Arc level (Fig. 5b), and the BDNF secretion (Fig. 5c) was observed without affecting the basal levels of all of them.

### The Dopamine D1-like Receptor Activation is Critical for p-CREB^S133^ and Arc Location

**Fig. 6:**
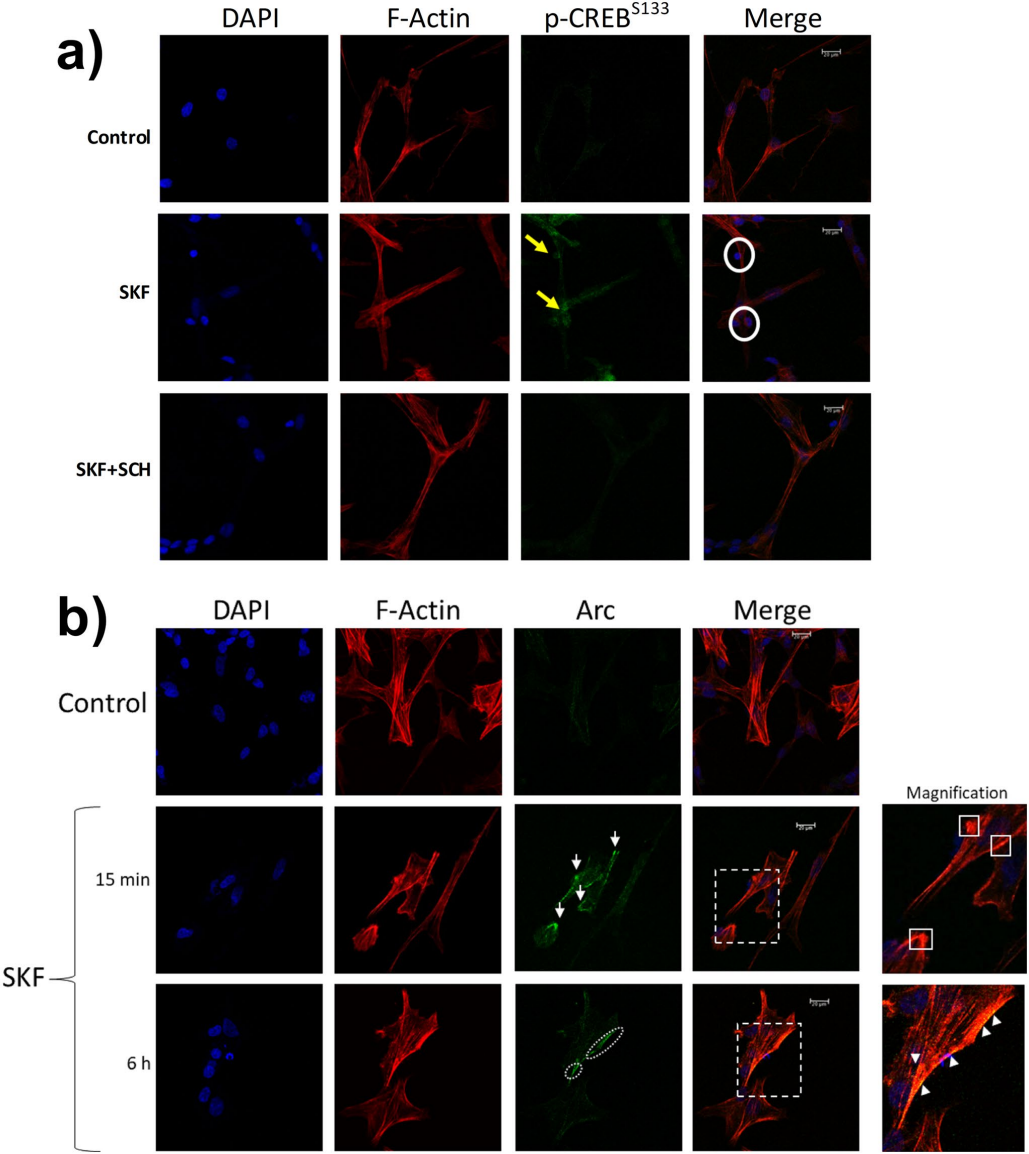
*Effects of dopamine D1-like receptor on p-CREB*^*S133*^*and Arc location*. After blocking (or not) the D1-like receptor, the cells were stimulated with SKF-38393 (10 µM) at indicated times. (**a**) After thirty minutes of stimulation, the p-CREB^S133^ location was immunodetected with an antibody against phosphorylated CREB in Ser^− 133^ (green); the F-actin was labeled with TRITC-phalloidin (red), and the nucleus was stained by DAPI (blue). For SKF-38,393 (10 µM), the yellow arrowheads indicate the prominent location of p-CREB^S133^ concurring in the nucleus visualized by the white circles in the merge column. (**b**) After fifteen minutes or six hours of stimulation, the Arc location was detected by immunofluorescence labeling Arc protein (green); the F-actin was labeled using TRITC-phalloidin (red); the nucleus was stained by DAPI (blue). An area (white dotted square) was magnified. The white arrow/squares point out the clusters of Arc near the cell membrane and lamellipodial F-actin structures without colors overlapping in merge at 0.25 h. In contrast, at 6 h, the white arrowheads point out the yellow dots corresponding to Arc location (green) overlapping with F-actin (red)

Dopamine D1-like receptor activation induced a remarkable nuclear location of p-CREB^S133^ at 0.5 h, compared to the scarce cytoplasmic and nuclear location of the non-activated control (Fig. 6a). Notably, the presence of SCH-23390 inhibited the nuclear location of p-CREB^S133^ induced by SKF-38393 (Fig. 6a). These results corroborate that dopamine D1-like receptor activation specifically induces CREB^S133^ phosphorylation at the nuclear level.

The Arc location was determined at times corresponding to both peaks (0.25 and 6 h). Compared to the non-stimulated control, the dopamine D1-like receptor activation induced Arc location as clusters near the cell membrane. However, only at 6 h Arc colocalizes with regions rich in lamellar F-actin compared to the 0.25 h Arc peak (Fig. 6b). As occurred with protein, SCH-23390 inhibited the effects of SKF-38393 on Arc (Fig. 1S in Online Resource 1).

Additionally, we recorded the morphological changes in cell morphology induced by dopamine D1-like receptor activation, as well as by the antagonist of the dopamine D1-like receptor (Fig. 2aS in Online Resource 1). The activation of the dopamine D1-like receptor with SKF-38393 did not induce an apparent change in cell morphology or neurite length compared to non-activated control cells. Remarkably, blocking the dopamine D1-like receptor with SCH-23390 antagonist produced a dramatic retraction of neurites compared to the non-activated control and SKF-38393 stimulation groups without affecting the viability cell in all conditions compared to the control group (Fig. 2bS in Online Resource 1).

### Discussion

The cognitive and executive functions of the brain are orchestrated by distinct neurotransmitters through their receptors, modulating the production and function of synaptic proteins [20]. Information about how dopamine D1-like receptor activation regulates the production of some relevant synaptic proteins is scarce, even though the evidence suggests that dopamine D1-like receptor activity is essential to several forms of synaptic plasticity [2, 3]​. The present study was focused on determining the effects of dopamine D1-like receptor activation on CREB, Arc, and BDNF, particularly on CREB activation and BDNF secretion temporality, as well as spatiotemporal induction of Arc.

### RA-treated SH-SY5Y Cells as a Study Model

Our study used RA-treated SH-SY5Y, which was previously reported as a suitable cell model for in vitro neurobiological studies, and it expresses the functional DA receptors [21, 22]. It is known that BDNF is secreted by mature neurons in in vivo physiological conditions [23]. The results did not show changes in the DRD1 protein expression in RA-differentiated SH-SY5Y compared to the undifferentiated cells. However, the morphological changes and increasing expression of Syn, p-CREB^S133^, and Arc and BDNF secretion supported previous studies reporting that RA-treated SH-SY5Y cells are a suitable cell model in our study.

### CREB as a Target of Dopamine D1-like Receptor Activation

As was expected, the maximum CREB activation (0.5 h) concurred with the maximum PKA activity (0.5 h), supporting that the PKA/CREB signaling axis is activated in response to dopamine D1-like receptor activation. Because CREB is the effector molecule in this axis, the changes in its activation over time were studied. CREB is a master regulator of *Arc*, *BDNF*, *c-fos*, *c-jun*, and other *IEG*s involved in cognitive functions [24]​​. CREB is activated by PKA among different kinds of kinases [25]. In the brain, CREB activity is induced via anionic channel activation, AMPA or NMDA receptors, and the dopamine D1-like receptor [6]. Our results indicate that the level of p-CREB^S133^ is dependent on dopamine D1-like receptor activation. The augment was *transient*, reaching the maximum level at 0.5 h. It has been suggested that the duration of phosphorylation of CREB depends on the received input and is critical to the subsequent expression of IEGs [26, 27]. The *c-fos* gene induced through D1/D5 receptors or via L-type Ca^2+^ channels correlated with *sustained* CREB phosphorylation but not with its *transient* phosphorylation [28]. However, growth hormone elicits *c-fos* expression associated with *transient* phosphorylation [29]. These data suggest that the distinctive effect depends on the input received.

Other pathways related to regulating sustained CREB phosphorylation in SH-SY5Y cells are phosphoinositide hydrolysis and Ca2 + mobilization [30]. t has been reported that the dopamine D1-like receptor activation by SKF-38393 increased the production of inositol phosphate in brain slices of wild-type mice but not in dopamine D5 receptor knockout, suggesting a role for DRD5 in phosphoinositide hydrolysis [31]. It has been reported that the mRNA of the *drd5* is expressed in SH-SY5Y cells [32]. At this concern, the potential DRD5 role, via phosphoinositide hydrolysis and Ca2 + mobilization, acting in parallel or independent of the PKA activation observed in our study, deserves to be evaluated.

On the other hand, the timing of CREB phosphorylation has behavioral implications. T*ransient* CREB phosphorylation has been reported in striatal neurons, where emotion and habit formation (decision-making) occur. In contrast, *sustained* CREB phosphorylation is recorded in limbic-related structures that process and regulate emotion, memory, and learning [10]. Further research is needed to determine the IEGs, parallel or independent pathways phosphorylating to CREB, and the neurophysiological functions derived from *transient* CREB phosphorylation induced by dopamine D1-like receptor activation.

### Dopamine D1-like Receptor-induced Local Synthesis of Arc

Arc participates in memory/learning formation through hippocampal long-term potentiation (LTP) and synaptic processes associated with dopamine D1-like receptor activation [3, 33]. However, its regulation from transcription, translation, and function has yet to be fully understood. Our results showed that dopamine D1-like receptor stimulation induced two transient spikes of Arc protein, an early increase (0.25 h) and a late increase (6 h). At both times, the location of Arc was near the cell membrane, forming clusters. However, at 6 h, the Arc signal was co-located with the F-actin signal, suggesting that dopamine D1-like receptor stimulation induces Arc spikes in specific subcellular regions depending on time. The early transient peak of Arc has yet to be reported. Arc’s mRNA is known to be coupled with a ribonucleoprotein complex and transported to specific subcellular sites, forming subcellular hubs of Arc mRNA, where it remains until a neuronal stimulus input promotes a rapid translation of the Arc mRNA/ribonucleoprotein complex [8]​. It has been proposed that these events are modulated by a process known as nonsense-mediated RNA decay (NMD), whose purpose is to eliminate certain kinds of RNA and is mainly orchestrated by eukaryotic translation initiation factor 4AIII [34]​. The Arc mRNA is a natural target of the 4AIII. In our study, the early transient peak of Arc may be related to a local translation of preexistent Arc mRNA, followed by a quick decay in protein level underlying the NMD process induced by dopamine D1-like receptor activation. The transient augment of Arc at 6 h could correspond to a second round of Arc translation depending on p-CREB^S133^ induced by intracellular pathways associated with dopamine D1-like receptor activation. It was shown that tetrodotoxin induced a second cycle of Arc translation in specific subcellular hotspots, operated by a feedback mechanism, where new proteins of the first cycle restart a second process of Arc transcription [35].

Nevertheless, this second cycle of Arc translation seems to depend on neuronal input; stimulation of muscarinic, cholinergic, and glutamate receptors induces gradual but sustained Arc protein augment [36, 37]. These data contrast with the fluctuant behavior shown in the present study for Arc in response to the activation of the dopamine D1-like receptor. The Arc decreasing after early and late peak takes neurophysiological relevance. In knock-in mice where the Arc ubiquitination sites were mutated, it was shown that the abnormal persistence of Arc protein level over time was associated with a failure in cognitive flexibility [11]. In this context, our results suggest that the dopamine D1-like receptor induces a particular dynamic in Arc protein induction with a specific timing; however, the meaning of the temporal expression on neurophysiological functions remains to be evaluated.

The late spike (6 h) of Arc protein, with subcellular colocalization with F-actin-rich regions, could be relevant to synaptic processes like LTP and Long-Term Depression (LTD). In response to stimulation with neurotransmitters, the dendritic spines undergo restructuring, involving morphological changes in actin cytoskeleton rearrangement [38]. As a coadjutant factor to actin polymerization dynamics, Arc interacts with actin-binding proteins, indirectly contributing to the regulation of neurotransmitter receptors’ enrichment in the cell membrane [7], which are necessary for LTP and LTD. The Arc protein induction, dependent on dopamine D1-like receptor activation, could be a relevant player for crosstalk between other neurotransmitter-induced synaptic events, which involve actin cytoskeleton remodeling in specific sites. Our study provides new insights into the role of dopamine D1-like receptors on Arc levels, the timing of expression, and their specific subcellular location to potentially participate in distinct neuronal function.

### BDNF Secretion by Dopamine D1-like Receptor Activation

The BDNF structure undergoes sequential modifications throughout its maturation process, resulting in pre-pro-BDNF, pro-BDNF, and mature BDNF [9]. Only mature BDNF is secreted from neuronal cells and acts at autocrine and paracrine levels via tropomyosin-related kinase B receptor (TrkB) [39]. How dopamine D1-like receptor activity contributes to BDNF regulation has yet to be fully elucidate. To our knowledge, this is the first study showing how dopamine D1-like receptor stimulation induces the secretion of mature BDNF with time-dependent fluctuations. Our results showed that after dopamine D1-like receptor activation, the maximum increase of BDNF secretion was reached at 6 h, partly correlating with intracellular BDNF protein in rat brain tissue stimulated with dopamine through the dopamine D1-like receptor [40]. This data suggests that the dopamine D1-like receptor activation regulates intracellular and secreted BDNF. The BDNF secretion decreased below the baseline at 24 h of dopamine D1-like receptor activation. The meaning and mechanism of late BDNF decreasing, dependent on dopamine D1-like receptor, has not been determined. It is known that the secreted BDNF bounds to the TrkB receptor, and the BDNF-TrkB complex is endocytosed via an endosomal pathway eliciting signaling events related to survival and synaptic and cognitive function [23, 41, 42]. Our results on BDNF levels at 24 h align with previous results suggesting that extracellular BDNF is internalized via BDNF-TrkB complex recycling in striatal neurons [43].

### Regulation of Neurite Extension by the Dopamine D1-like Receptor

Neurites are structures that are essential for establishing synaptic contact among neurons that are far from each other. In processes like neurodevelopment, neurites will become axonal or dendritic structures [44], and DA regulates neuronal connectivity development via its receptors [45, 46]. Our results suggest that the dopamine D1-like receptor is relevant in maintaining neurite extension of RA-treated SH-SY5Y since neurite retraction occurred when the dopamine D1-like receptor was blocked. Previously, it was demonstrated that dopamine D1-like receptor induces growth cone formation and arborization in rat striatum [46]. Conversely, DA can also decrease the neurite length in rat cortical neurons [45]. This paradox may depend on DA tonicity and availability in the synaptic cleft; the DA will activate the dopamine D1-like receptor or the D2-like receptor with contrasting effects, high and low doses of DA, respectively [47]. There is contradictory evidence about the D2-like receptor expression and dopamine secretion after the differentiation process in SH-SY5Y cells [48, 49]. If the cells were producing and releasing dopamine and expressing D2-like receptors, dopamine would activate these receptors, inducing neurite retraction. However, in our study, the control SH-SY5Y cells RA-differentiated showed neurite extension (Fig. 2S). These results suggest that in case of occurring, the dopamine (if it is secreted) and D2 receptor activity do not affect the neurite extension. These results support that SCH-23390 induces neurite retraction by antagonizing the D1 receptor effects.

In our study, since a dopamine D1-like receptor agonist was used (SKF-38393), a bimodal pattern typical of DA was avoided, allowing us to hypothesize a potential role specifically for the dopamine D1-like receptor on neuronal projections regulation, maybe through BDNF. The dopamine D1-like receptor antagonist inhibited the BDNF secretion concomitantly with neurite retraction. One of the roles of BDNF is to regulate neuronal morphology, like neurites, dendritic, and growth cone formation, during the neurodevelopment process [9]. Previously, it was demonstrated that deleting BDNF expression in hippocampal neurons produces failure in dendrite development [39]. In another study, the treatment with BDNF enhanced hippocampal neurons’ number and neurite length [50]. Our results suggest that the dopamine D1-like receptor activation induces BDNF secretion, which is probably involved, at least partly, in neurite extension regulation.

**Fig. 7:**
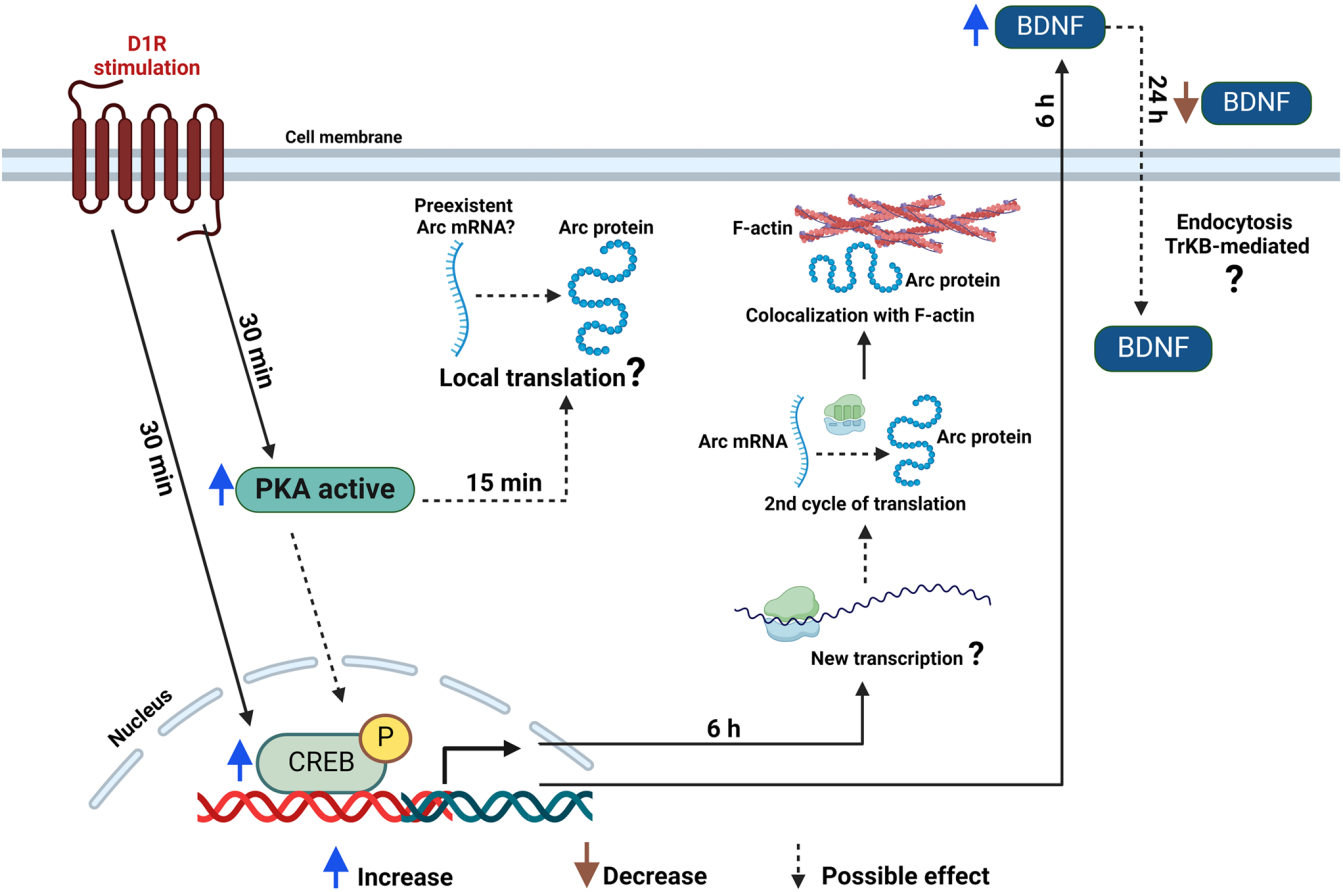
Schematical representation of dynamic changes by dopamine D1-like receptor activation on p-CREB ^S133^, Arc, BDNF. PKA is activated once the dopamine D1-like receptor is stimulated, and CREB is phosphorylated (*p-CREB*^*S133*^). Arc protein is increased at 15 min, suggesting preexistent mRNA translation; a second increase at 6 suggests a second cycle of transcription and translation. BDNF secretion fluctuates over time, suggesting that in addition to stimulation of secretion, at 24 h, the dopamine D1-like receptor would induce the BDNF endocytosis (scheme was created with BioRender.com)

In conclusion, dopamine D1-like receptor stimulation transiently induces CREB activation, the Arc protein expression in a spatiotemporal dynamic manner, fluctuant changes in BDNF secretion depending on the time, and regulates the neurites extension (Fig. 7). This study provides new insights into the role of the dopamine D1-like receptor on CREB, Arc, and BDNF, critical players for synaptic functions. The implication and relevance of the dopamine D1-like receptor for regulating distinct processes associated with cognitive functions remained for further studies.

## Electronic Supplementary Material

Below is the link to the electronic supplementary material.

Supplementary Material 1

## Data Availability

No datasets were generated or analysed during the current study.

## References

[1] Beaulieu J-M, Gainetdinov RR (2011) The physiology, signaling, and pharmacology of dopamine receptors. Pharmacol Rev 63 182 LP– 217. 10.1124/pr.110.002642 10.1124/pr.110.002642 21303898

[2] Daba Feyissa D, Sialana FJ, Keimpema E et al (2019) Dopamine type 1- and 2-like signaling in the modulation of spatial reference learning and memory. Behav Brain Res 362:173–180. 10.1016/J.BBR.2019.01.028 30659847 10.1016/j.bbr.2019.01.028

[3] Granado N, Ortiz O, Suárez LM et al (2008) D1 but not D5 dopamine receptors are critical for LTP, spatial learning, and LTP-Induced arc and zif268 expression in the Hippocampus. Cereb Cortex 18:1–12. 10.1093/cercor/bhm026 17395606 10.1093/cercor/bhm026

[4] Jones-Tabah J, Mohammad H, Paulus EG et al (2022) The signaling and pharmacology of the dopamine D1 receptor. Front Cell Neurosci 15:1–28. 10.3389/fncel.2021.806618 10.3389/fncel.2021.806618 PMC8801442 35110997

[5] Steven A, Friedrich M, Jank P et al (2020) What turns CREB on? And off? And why does it matter? Cell Mol Life Sci 77:4049–4067. 10.1007/s00018-020-03525-8 32347317 10.1007/s00018-020-03525-8 PMC7532970

[6] Ortega-Martínez S (2015) A new perspective on the role of the CREB family of transcription factors in memory consolidation via adult hippocampal neurogenesis. Front Mol Neurosci 8:1–12. 10.3389/fnmol.2015.00046 26379491 10.3389/fnmol.2015.00046 PMC4549561

[7] Nikolaienko O, Patil S, Eriksen MS, Bramham CR (2018) Arc protein: a flexible hub for synaptic plasticity and cognition. Semin Cell Dev Biol 77:33–42. 10.1016/J.SEMCDB.2017.09.006 28890419 10.1016/j.semcdb.2017.09.006

[8] Bramham CR, Alme MN, Bittins M et al (2010) The Arc of synaptic memory. Exp Brain Res 200:125–140. 10.1007/s00221-009-1959-2 19690847 10.1007/s00221-009-1959-2 PMC2803749

[9] Kowiański P, Lietzau G, Czuba E et al (2018) BDNF: a key factor with multipotent impact on Brain Signaling and synaptic plasticity. Cell Mol Neurobiol 38:579–593. 10.1007/s10571-017-0510-4 28623429 10.1007/s10571-017-0510-4 PMC5835061

[10] Liu FUC, Graybiel AM (1998) Region-dependent dynamics of cAMP response element-binding protein phosphorylation in the basal ganglia. Proc Natl Acad Sci U S A 95:4708–4713. 10.1073/pnas.95.8.4708 9539803 10.1073/pnas.95.8.4708 PMC22555

[11] Wall MJ, Collins DR, Chery SL et al (2018) The temporal dynamics of Arc expression regulate cognitive flexibility. Neuron 98:1124–1132e7. 10.1016/J.NEURON.2018.05.012 29861284 10.1016/j.neuron.2018.05.012 PMC6030446

[12] Anderson F, Siller J, Barral EM J (2011) Disorders of Protein Biogenesis and Stability. Protein Pept Lett 18:110–121. 10.2174/092986611794475075 21121895 10.2174/092986611794475075

[13] Mahapatra KK, Panigrahi DP, Praharaj PP et al (2019) Molecular interplay of autophagy and endocytosis in human health and diseases. Biol Rev 94:1576–1590. 10.1111/brv.12515 30989802 10.1111/brv.12515

[14] Encinas M, Iglesias M, Liu Y et al (2000) Sequential treatment of SH-SY5Y cells with retinoic acid and brain-derived neurotrophic factor gives rise to fully differentiated, neurotrophic factor-dependent, human neuron-like cells. J Neurochem 75:991–1003. 10.1046/j.1471-4159.2000.0750991.x 10936180 10.1046/j.1471-4159.2000.0750991.x

[15] Chin-Chan M, Segovia J, Quintanar L et al (2015) Mercury reduces the enzymatic activity of neprilysin in differentiated SH-SY5Y cells. Toxicol Sci 145:128–137. 10.1093/toxsci/kfv037 25673500 10.1093/toxsci/kfv037 PMC4833038

[16] Mosmann T (1983) Rapid Colorimetric Assay for Cellular Growth and Survival: application to proliferation and cytotoxicity assays. J Lmmunological Methods 65:55–63. 10.1039/c6ra17788c 10.1016/0022-1759(83)90303-4 6606682

[17] Hernández AJA, Reyes VL, Albores-García D et al (2018) MeHg affects the activation of FAK, src, Rac1 and Cdc42, critical proteins for cell movement in PDGF-stimulated SH-SY5Y neuroblastoma cells. Toxicology 394:35–44. 10.1016/j.tox.2017.11.019 29197552 10.1016/j.tox.2017.11.019

[18] Rodriguez-Monterrosas C, Díaz-Aragon R, Leal-Orta E et al (2018) Insulin induces an EMT-like process in mammary epithelial cells MCF10A. J Cell Biochem 119:4061–4071. 10.1002/jcb.26582 29236310 10.1002/jcb.26582

[19] Reyes-Vázquez L, Hernández AJA, Calderón-Aranda ES (2020) Role of aromatase activation on sodium arsenite-induced proliferation, migration, and invasion of MDA-MB-231 and MDA-MB-453 breast cancer cell lines. Toxicology 437:152440. 10.1016/j.tox.2020.152440 32197950 10.1016/j.tox.2020.152440

[20] Robbins TW (2011) Cognition: the ultimate brain function. Neuropsychopharmacology 36:1–2. 10.1038/npp.2010.171 21116247 10.1038/npp.2010.171 PMC3055511

[21] Teppola H, Sarkanen JR, Jalonen TO, Linne ML (2016) Morphological differentiation towards neuronal phenotype of SH-SY5Y Neuroblastoma cells by Estradiol, retinoic acid and cholesterol. Neurochem Res 41:731–747. 10.1007/s11064-015-1743-6 26518675 10.1007/s11064-015-1743-6 PMC4824837

[22] Xie HR, Hu L, Sen, Li GY (2010) SH-SY5Y human neuroblastoma cell line: in vitro cell model of dopaminergic neurons in Parkinson’s disease. Chin Med J (Engl) 123:1086–1092. 10.3760/cma.j.issn.0366-6999.2010.08.021 20497720

[23] Lu B (2003) BDNF and activity-dependent synaptic modulation. Learn Memory 10:86–98. 10.1101/lm.54603 10.1101/lm.54603 PMC5479144 12663747

[24] Kaldun JC, Sprecher SG (2019) Initiated by CREB: Resolving Gene Regulatory Programs in Learning and Memory: switch in cofactors and transcription regulators between Memory Consolidation and Maintenance Network. BioEssays 41:1–14. 10.1002/bies.201900045 31237359 10.1002/bies.201900045

[25] Zhang H, Kong Q, Wang J et al (2020) Complex roles of cAMP–PKA–CREB signaling in cancer. Exp Hematol Oncol 9:1–13. 10.1186/s40164-020-00191-1 33292604 10.1186/s40164-020-00191-1 PMC7684908

[26] Nijholt I, Blank T, Ahi J, Spiess J (2002) In vivo CREB phosphorylation mediated by dopamine and NMDA receptor activation in mouse hippocampus and caudate nucleus. Brain Res Gene Expr Patterns 1:101–106. 10.1016/s1567-133x(01)00020-5 15018806 10.1016/s1567-133x(01)00020-5

[27] Soleimanpour E, Bergado Acosta JR, Landgraf P et al (2021) Regulation of CREB phosphorylation in nucleus accumbens after relief conditioning. Cells 10:1–15. 10.3390/cells10020238 10.3390/cells10020238 PMC7912172 33530478

[28] Liu FC, Graybiel AM (1996) Spatiotemporal dynamics of CREB phosphorylation: transient versus sustained phosphorylation in the developing striatum. Neuron 17:1133–1144. 10.1016/S0896-6273(00)80245-7 8982161 10.1016/s0896-6273(00)80245-7

[29] Cui TX, Kwok R, Schwartz J (2008) Cooperative regulation of endogenous cAMP-response element binding protein and CCAAT/enhancer-binding protein β in GH-stimulated c-fos expression. J Endocrinol 196:89–100. 10.1677/JOE-07-0169 18180320 10.1677/JOE-07-0169

[30] Rosethorne EM, Nahorski SR, Challiss RAJ (2008) Regulation of cyclic AMP response-element binding-protein (CREB) by Gq/11-protein-coupled receptors in human SH-SY5Y neuroblastoma cells. Biochem Pharmacol 75:942–955. 10.1016/j.bcp.2007.10.015 18036509 10.1016/j.bcp.2007.10.015 PMC2593902

[31] Sahu A, Tyeryar KR, Vongtau HO et al (2009) D 5 dopamine receptors are required for dopaminergic activation of phospholipase C. Mol Pharmacol 75:447–453. 10.1124/mol.108.053017 19047479 10.1124/mol.108.053017 PMC2684903

[32] Zhou J, Peng C, Li Q Dopamine Homeostasis Imbalance and Dopamine, Receptors-Mediated et al (2022) AC/cAMP/PKA Pathway Activation are Involved in Aconitine-Induced Neurological Impairment in Zebrafish and SH-SY5Y Cells. Front Pharmacol 13:1–21. 10.3389/fphar.2022.837810 10.3389/fphar.2022.837810 PMC8971779 35370746

[33] Saito N, Tainaka K, Macpherson T et al (2020) Neurotransmission through dopamine D1 receptors is required for aversive memory formation and Arc activation in the cerebral cortex. Neurosci Res 156:58–65. 10.1016/J.NEURES.2020.04.006 32380131 10.1016/j.neures.2020.04.006

[34] Giorgi C, Yeo GW, Stone ME et al (2007) The EJC factor eIF4AIII modulates synaptic strength and neuronal protein expression. Cell 130:179–191. 10.1016/J.CELL.2007.05.028 17632064 10.1016/j.cell.2007.05.028

[35] Das S, Lituma PJ, Castillo PE, Singer RH (2023) Maintenance of a short-lived protein required for long-term memory involves cycles of transcription and local translation. Neuron 111:2051–2064.e6. 10.1016/J.NEURON.2023.04.005 37100055 10.1016/j.neuron.2023.04.005 PMC10330212

[36] Chen T, Zhu J, Yang LK et al (2017) Glutamate-induced rapid induction of Arc/Arg3.1 requires NMDA receptor-mediated phosphorylation of ERK and CREB. Neurosci Lett 661:23–28. 10.1016/J.NEULET.2017.09.024 28919534 10.1016/j.neulet.2017.09.024

[37] Soulé J, Alme M, Myrum C et al (2012) Balancing Arc Synthesis, mRNA decay, and proteasomal degradation: MAXIMAL PROTEIN EXPRESSION TRIGGERED BY RAPID EYE MOVEMENT SLEEP-LIKE BURSTS OF MUSCARINIC CHOLINERGIC RECEPTOR STIMULATION. J Biol Chem 287:22354–22366. 10.1074/JBC.M112.376491 22584581 10.1074/jbc.M112.376491 PMC3381195

[38] Chazeau A, Giannone G (2016) Organization and dynamics of the actin cytoskeleton during dendritic spine morphological remodeling. Cell Mol Life Sci 73:3053–3073. 10.1007/s00018-016-2214-1 27105623 10.1007/s00018-016-2214-1 PMC11108290

[39] Wang L, Chang X, She L et al (2015) Autocrine Action of BDNF on Dendrite Development of Adult-born hippocampal neurons. J Neurosci 35:8384–8393. 10.1523/JNEUROSCI.4682-14.2015 26041908 10.1523/JNEUROSCI.4682-14.2015 PMC6605324

[40] Williams SN, Undieh AS (2009) Dopamine D1-like receptor activation induces brain-derived neurotrophic factor protein expression. NeuroReport 20:606–610. 10.1097/WNR.0b013e32832a0a98 19295451 10.1097/WNR.0b013e32832a0a98 PMC2834182

[41] Moya-Alvarado G, Tiburcio-Felix R, Ibáñez MR et al (2023) BDNF/TrkB signaling endosomes in axons coordinate CREB/mTOR activation and protein synthesis in the cell body to induce dendritic growth in cortical neurons. Elife 12. 10.7554/eLife.77455 10.7554/eLife.77455 PMC9977295 36826992

[42] Piromalli Girado D, Miranda M, Giachero M et al (2023) Endocytosis is required for consolidation of pattern-separated memories in the perirhinal cortex. Front Syst Neurosci 17:1–13. 10.3389/fnsys.2023.1043664 10.3389/fnsys.2023.1043664 PMC9995888 36911226

[43] Iwakura Y, Nawa H, Sora I, Chao MV (2008) Dopamine D1 receptor-induced signaling through TrkB receptors in Striatal Neurons. J Biol Chem 283:15799–15806. 10.1074/JBC.M801553200 18381284 10.1074/jbc.M801553200 PMC2414263

[44] Gallo G (2013) Mechanisms underlying the initiation and Dynamics of neuronal Filopodia: from neurite formation to synaptogenesis. Int Rev Cell Mol Biol 301:95–156. 10.1016/B978-0-12-407704-1.00003-8 23317818 10.1016/B978-0-12-407704-1.00003-8

[45] Lankford KL, DeMello FG, Klein WL (1988) D1-type dopamine receptors inhibit growth cone motility in cultured retina neurons: evidence that neurotransmitters act as morphogenic growth regulators in the developing central nervous system. Proceedings of the National Academy of Sciences 85:4567–4571. 10.1073/pnas.85.12.4567-a 10.1073/pnas.85.12.4567-a PMC280472 3380807

[46] Reinoso BS, Undie AS, Levitt P (1996) Dopamine receptors mediate differential morphological effects on cerebral cortical neurons in vitro. J Neurosci Res 43:439–453. 10.1002/(SICI)1097-4547(19960215)43:4%3C439::AID-JNR5%3E3.0.CO;2-G 10.1002/(SICI)1097-4547(19960215)43:4<439::AID-JNR5>3.0.CO;2-G 8699530

[47] Baik J-H (2013) Dopamine signaling in reward-related behaviors. Front Neural Circuits 7 10.3389/fncir.2013.00152 PMC3795306 24130517

[48] Mastroeni D, Grover A, Leonard B et al (2009) Microglial responses to dopamine in a cell culture model of Parkinson’s disease. Neurobiol Aging 30:1805–1817. 10.1016/j.neurobiolaging.2008.01.001 18325635 10.1016/j.neurobiolaging.2008.01.001 PMC2762863

[49] Magalingam KB, Radhakrishnan AK, Somanath SD et al (2020) Influence of serum concentration in retinoic acid and phorbol ester induced differentiation of SH-SY5Y human neuroblastoma cell line. Mol Biol Rep 47:8775–8788. 10.1007/s11033-020-05925-2 33098048 10.1007/s11033-020-05925-2

[50] Ogata K, Shintani N, Hayata-Takano A et al (2015) PACAP enhances Axon Outgrowth in cultured hippocampal neurons to a comparable extent as BDNF. PLoS ONE 10:e0120526. 10.1371/journal.pone.0120526 25807538 10.1371/journal.pone.0120526 PMC4373823

