# Supplementary material for: Dopamine D1-Like Receptor Stimulation Induces CREB, Arc, and BDNF Dynamic Changes in Differentiated SH-SY5Y Cells
Source: Neurochem Res. 2024 Nov 27;50(1):35. doi: 10.1007/s11064-024-04293-8 (PMC11602804; doi:10.1007/s11064-024-04293-8)
Supplement: Supplementary file 1 — Supplementary Material 1 [file 11064_2024_4293_MOESM1_ESM.pdf]

Supplementary Information

Dopamine 1-like receptor stimulation induces dynamic changes in CREB, Arc, and BDNF in differentiated SH-SY5Y cells

Omar B. Rivera-Maya, Christian D. Ortiz-Robles, José R. Palacios-Valladares, Emma S. Calderón-Aranda*

Center for Research and Advanced Studies of the National Polytechnic Institute, Department of Toxicology, Mexico City, Mexico.

*Corresponding author: Emma S. Calderón Aranda

scalder@cinvestav.mx

Included material:

Fig. 1S Effects of the D1R-like receptor blockade on Arc location.

Fig. 2S Morphologic changes induced by the agonist and a blocker of the D1-like receptor.

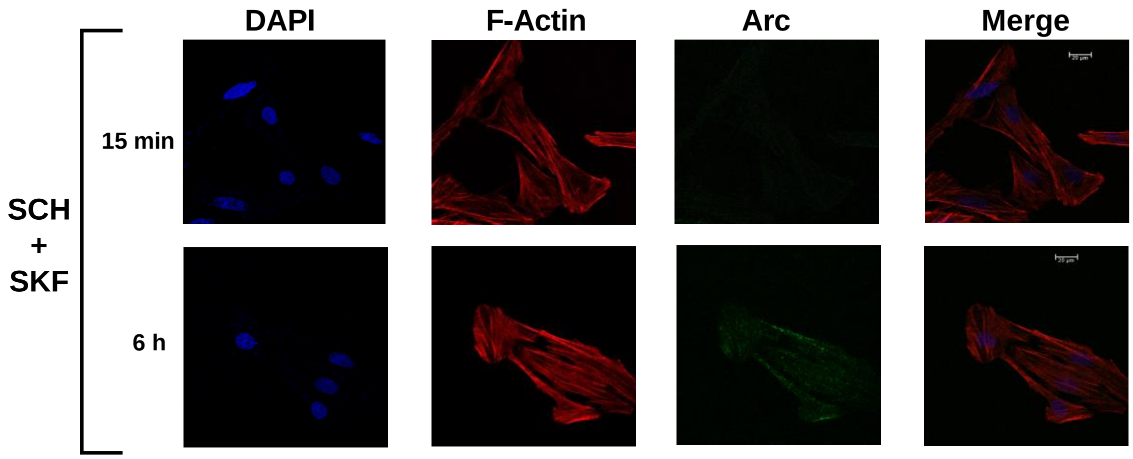

Fig. 1S *Effects of the D1R-like receptor blockade on Arc location*. After blocking of D1-like receptor, the cells were stimulated for fifteen minutes or six hours, the Arc location was detected by immunofluorescence labeling Arc protein (green); the F-actin was labeled using TRITC-phalloidin (red); the nucleus was stained by DAPI (blue). The absence of clusters of Arc and its colocalization is observed in both times by SCH treatment.

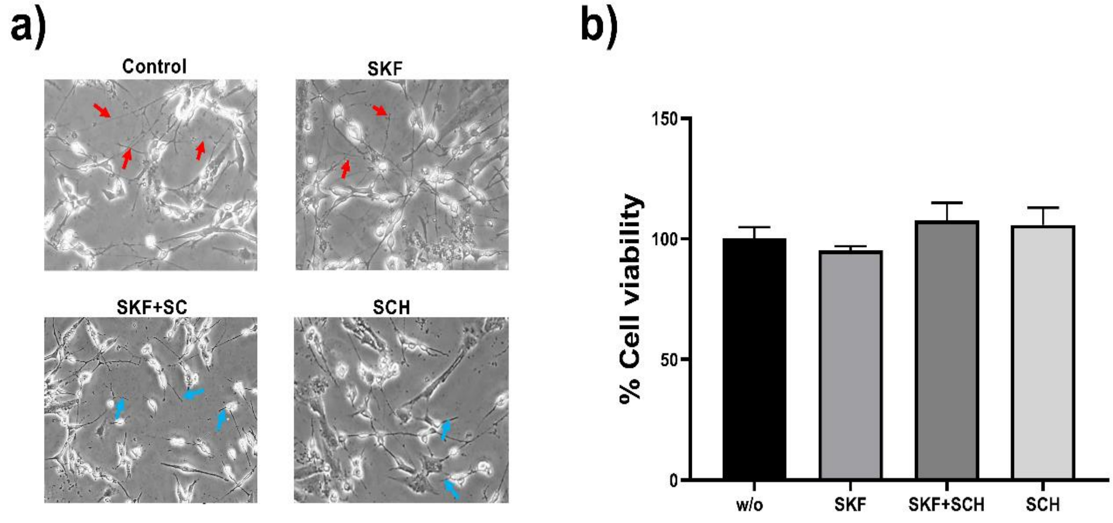

Fig. 2S M*orphologic changes induced by the agonist and a blocker of the D1-like receptor.* a) The micrographs by phase contrast microscope show numerous and lengthy processes (red arrows) arising from the cell's soma in control and SKF cultures, blockage of D1-like receptor by SCH induced neurite retraction in SKF+SCH and SCH cultures (blue arrows); b) the viability cell evaluated by MTT after 12 h of non-treatment, SKF, SKF plus SCH, or SCH treatment. A One-Way ANOVA test followed by Tukey´s post hoc test
